# Reporting behaviour change interventions: do the behaviour change technique taxonomy v1, and training in its use, improve the quality of intervention descriptions?

**DOI:** 10.1186/s13012-016-0448-9

**Published:** 2016-06-07

**Authors:** Caroline E. Wood, Wendy Hardeman, Marie Johnston, Jill Francis, Charles Abraham, Susan Michie

**Affiliations:** 1 UCL Centre for Behaviour Change, University College London, 1-19 Torrington Place, London, WC1E 7HB UK; 2 School of Health Sciences, University of East Anglia, Norwich Research Park, Norwich, Norfolk NR4 7TJ UK; 3 Institute of Applied Health Sciences, College of Life Science and Medicine, University of Aberdeen, 2nd Floor, Health Sciences Building, Aberdeen, AB25 2ZD UK; 4 School of Health Sciences, City University London, Myddelton Street Building, Northampton Square, London, EC1V 0HB UK; 5 Psychology Applied to Health Group, University of Exeter Medical School, University of Exeter, St Luke’s Campus, Exeter, EX1 2LU UK

**Keywords:** Behaviour change, Reporting interventions, Taxonomy

## Abstract

**Background:**

Behaviour change interventions are likely to be reproducible only if reported clearly. We assessed whether the behaviour change technique taxonomy version 1 (BCTTv1), with and without training in identifying BCTs, improves the clarity and replicability of written reports of observed behaviour change interventions.

**Methods:**

Three studies assessed effects of using and training in the use of BCTTv1 on the clarity and replicability of intervention descriptions written after observing videos of smoking cessation interventions. Study 1 examined the effects of using and not using BCTTv1. Study 2 examined the effects of using BCTTv1 and training in use of BCTTv1 compared no use and no training. Study 3 employed a within-group design to assess change in descriptions written before and after training. One-hundred and 66 ‘writers’ watched videos of behaviour change interventions and wrote descriptions of the active components delivered. In all studies, the participants’ written descriptions were evaluated by (i) 12 ‘raters’ (untrained in BCTTv1) for clarity and replicability and (ii) 12 ‘coders’ (trained in BCTTv1) for reliability of BCT coding. Writers rated the usability and accessibility of using BCTTv1 to write descriptions.

**Results:**

Ratings of clarity and replicability did not differ between groups in study 1 (all *p*s > 0.05), were poorer for trained users in study 2 (all *p*s < 0.01) and improved following training in study 3 (all *p*s < 0.05). BCT identification was more reliable from descriptions written by trained BCTTv1 users (*p* < 0.05; study 2) but not simple use of BCTTv1 (*p* = 0.93; study 1) or by writers who had written a description without BCTTv1, before training (*p* = 0.50; study 3). Writers reported that using BCTTv1 was difficult but ‘useful’, ‘good’ and ‘desirable’ and that their descriptions would be clear and replicable (all means above mid-point of the scale).

**Conclusions:**

Effects of training to use BCTTv1 on the quality of written reports of observed interventions were mixed, with some suggestion of improved clarity and replicability of reporting in the within- (study 3) but not the between-group studies (studies 1 and 2). Potential benefits of using BCTTv1 may have been limited by the artificial nature and time constraints of the task.

**Electronic supplementary material:**

The online version of this article (doi:10.1186/s13012-016-0448-9) contains supplementary material, which is available to authorized users.

## Background

To improve implementation and replication of effective behaviour change interventions (BCIs), we need better methods to specify and report potentially ‘active ingredients’. Reviews show that precise details of interventions are often omitted from intervention descriptions [1] leaving readers unable to accurately understand, evaluate and/or replicate the intervention reported. Glasziou et al. [1] found that 67 % of pharmacological intervention descriptions were accurately described, compared with only 29 % of non-pharmacological intervention descriptions. A similar illustrative review found that, from a sample of 198 published studies, those reporting pharmacological interventions were significantly more likely to specify the active ingredients of the intervention in the title and abstract (88 and 95 %, respectively) than those reporting non-pharmacological interventions (51 and 71 %) [2].

Non-pharmacological interventions, such as technical procedures, rehabilitation, BCIs, and psychotherapy are often complex and involve several interacting components. Randomised controlled trials of non-pharmacological interventions account for approximately one in four publications of trials [3] and the majority of these are BCIs [2]. Guidance is available to aid transparent reporting of non-pharmacological interventions, yet specific guidance for reporting complex BCIs is lacking. The CONSORT (Consolidated Standards of Reporting Trials) statement for randomised trials of ‘non-pharmacological’ interventions recommends precise specification of trial processes, including some details of the delivery of interventions and ‘description of the different components of the intervention’ [3, 4]. However, CONSORT does not provide guidance about what this ‘description’ or ‘components’ should be. Davidson et al. [5] suggested that intervention components include who delivers the intervention, to whom, how often, for how long, in what format, in what context, and with what content. The TIDieR (Template for Intervention Description and Replication) checklist [6] expanded on this list, the CONSORT statement and other guidance including the SPIRIT statement (Standard Protocol items: Recommendations for Interventional Trials) [7]. The TIDieR checklist specifies the essential elements which should be reported for any interventions, including behavioural, surgical and pharmaceutical interventions. Good reporting requires writers to specify characteristics of interventions and their context such as mode of delivery, intervention intensity, target behaviour, target population, setting and active content [8]. The criteria in the TIDieR checklist, however, are mainly procedures for reporting delivery (‘mode of delivery’, such as face-to-face or the internet) rather than active content (e.g. goal setting).

There is a need for a tool which enables researchers, practitioners and policy makers to report the potentially active ingredients of interventions more clearly and precisely, using a common, shared language. This would enable effective interventions to be more effectively replicated (e.g. across settings such as health care versus community settings) and by other organisations (e.g. schools and the workplace).

If the quality of written intervention reports is to improve, writers from research, practice and policy need to become more skilled in recognising poor reporting in published articles and in using a methodology to improve their reporting of interventions. One method for improving the reporting of BCI content is to specify it in terms of its component behaviour change techniques (BCTs). BCTs are ‘the smallest components compatible with retaining the postulated ingredients i.e. the proposed mechanisms of change. They can be used alone or in combination with other BCTs’ [9, 10]. The specification of content by BCTs offers several advantages. Firstly, it enables more robust evidence synthesis about which BCTs are potentially effective within a complex intervention and the extent to which they vary as a function of contextual variables (e.g. setting, target population). Secondly, it promotes the accurate replication of RCTs and other study designs in research settings by precisely specifying both intervention and control conditions (e.g. ‘usual care’). Thirdly, it facilitates faithful implementation of effective interventions in research, practice and policy. Fourthly, linking BCTs with theories of behaviour change and theoretical constructs allows the investigation of mechanisms underlying any intervention effects.

In sum, the use of a tool which specifies BCTs will help researchers, practitioners and policy makers to report their interventions more precisely using a shared language, facilitating evidence synthesis and the roll-out of effective behaviour change interventions in practice and policy. Clearer reporting of interventions would mean that the BCTs in the intervention are reported in a way that makes them more reliably identifiable and more replicable [11, 12]. However, such a tool requires training those who use it to report the content of their interventions.

Building on definitions of techniques frequently observed in BCIs by Abraham and Michie [13], an extensive, hierarchically structured taxonomy of BCTs was developed: the behaviour change technique taxonomy version 1 (BCTTv1, [14]). The tool can be used reliably to identify techniques defined by BCTTv1 from intervention descriptions [15, 16]. Achieving good levels of reliability in BCT identification requires skill and training in using BCTTv1. We found that face-to-face workshops and distance group tutorials improved agreement with expert consensus about the *identification* of BCTs [16] and the *interpretation* of BCTs in written reports of BCIs (Johnston M, Johnston D, Wood C, Hardeman W, Francis J, Michie S. Communication of behaviour change interventions: can their reporting and interpretation be improved using the behaviour change technique taxonomy (v1)? Under Review), such as the descriptions of interventions in published journal articles. Whilst these studies showed that training in BCTTv1 improves agreement about the presence of BCTs in intervention descriptions, the potential of BCTTv1 to improve the *writing* of intervention descriptions has not been investigated.

In this paper, we examine the utility of BCTTv1 in terms of writing descriptions of *observed* behaviour change interventions. We investigate the effects of (i) using BCTTv1 (i.e. being provided with a copy and consulting it during the writing process) and (ii) training in using BCTTv1, on the clarity and replicability of written intervention descriptions. We used three study designs. Studies 1 and 2 used a randomised between-group design to estimate the effects of using BCTTv1, both with and without training, on the clarity (e.g. ease of understanding) and replicability (e.g. adequacy of information required to undertake a replication) of written intervention descriptions. Study 3 used a before-after, within-group design to estimate the effects of using BCTTv1 and training on the quality and replicability of written intervention descriptions.

In all three studies, participants watched videos of BCIs delivered in practice and were then asked to write a description of the BCIs. The studies involved three types of participants: ‘writers’, ‘raters’ and ‘coders’. Participants (the ‘writers’) watched the videos and were asked to write descriptions of the intervention they observed. The quality and replicability of the intervention descriptions were assessed by independent, untrained ‘raters’ who were not familiar with BCTTv1. In addition, we asked trained ‘coders’ to identify the BCTs from the intervention descriptions. We calculated agreement between coder pairs and compared BCT coding by coders with the consensus reached by experienced coders from the BCTT project team (‘developer consensus’). Finally, we asked all ‘writers’ to rate the usefulness of BCTTv1 for reporting BCIs. The research questions were

1. Are descriptions of observed BCIs generated by untrained writers using BCTTv1 (i) clearer and (ii) more replicable than descriptions written by untrained writers not using BCTTv1?
2. Are descriptions of observed BCIs generated by trained writers using BCTTv1 (i) clearer and (ii) more replicable than descriptions written by untrained writers not using BCTTv1?
3. Does the reliability of BCT identification differ for descriptions of observed BCIs written by (i) untrained writers not using BCTTv1, (ii) untrained writers using BCTTv1 and (iii) trained writers using BCTTv1?
4. How does the coding of ‘trained coders’ compare with that of developer consensus?
5. Do users report BCTTv1 to be a useful and acceptable tool for reporting the content of BCIs?

## Methods

### Design

Three studies were conducted to address the research questions (RQs) above. All studies involved participants watching a video of a BCI and then writing a short description of the BCI. Study 1 addressed RQ1 and was a randomised controlled trial (RCT) to assess the effects on quality of the intervention descriptions written with and without using BCTTv1, among participants who had not been trained in its use. A copy of BCTTv1 was provided to the experimental group but not the control group. A second RCT (study 2) compared the effects of writing intervention descriptions using BCTTv1 following training in its use with a control group who received neither BCTTv1 nor relevant training. Study 3 used a before-after, within-group design to assess change in writing before training, without using BCTTv1 and writing after training, using BCTTv1. Both studies 2 and 3 aimed to answer RQ2 and RQ3.

To counteract potential practice and order effects, we used two videos with the same target behaviour (smoking cessation) which were administered in a counterbalanced design (i.e. half of the writers were shown video 1 and then completed the writing task and the other half of the writers were shown video 2 and then completed the writing task).

For each study, the written descriptions were assessed in terms of their clarity and replicability, by a group of ‘raters’. To assess reliability and validity of BCT identification from the written descriptions, a separate group of ‘coders’ coded the descriptions for BCTs and this was compared with coding carried out by the BCTTv1 project team (‘developer consensus’) (RQ4). Writers rated the usefulness of BCTTv1 for reporting (RQ5).

### Materials

#### Videos

Two videos were used targeting smoking cessation, including a range of frequently observed and clearly delivered BCTs (e.g. social support (unspecified), feedback on behaviour). Both were approximately 9 min in duration and showed a smoking cessation practitioner delivering the intervention to a service user.

#### Consensus on BCTs delivered

To establish consensus about the presence of BCTs in the videos, four experienced BCT coders who had been involved in developing BCTTv1 (MJ, JF, SM and WH) independently coded both videos using BCTTv1. Each video was coded by two coders and discrepancies were discussed within each of the pairs. If a resolution was not obvious, the senior author SM and the study researcher (CW) reviewed the remaining discrepancies. Coding was then agreed by all coders. This process established ‘developer consensus’ about the presence/absence of 21 BCTs in video 1 and 15 BCTs in video 2 (see Additional file 1).

#### BCTTv1

Dependent on the study, writers were provided with a paper copy of BCTTv1 to refer to whilst writing their description (Michie et al. 2013).

### Participants

There were three types of participant: writers, raters and coders. Since they differed across studies, participant details are given separately for each study (see Table 1).

**Table 1:** Summary of participants in studies 1, 2 and 3

Study	Participants	*N*	Mean age	% UK	% doctorate	% practitioner	% use of BCTTv1		Expertise in BCIs Mean (SD)
							Code	Describe	
1	Writers	42	37.77	95	46	32	12	29	2.42 (1.20)
	Raters	4	30.50	100	0	100	0	0	2.00 (1.47)
	Coders	4	32.25	50	75	25	75	75	3.50 (0.37)
2	Writers	85	39.08	95	51	28	5	6	2.32 (1.09)
	Raters	4	32.50	100	0	100	0	0	2.00 (1.22)
	Coders	4	31.75	100	100	25	100	100	3.44 (0.59)
3	Writers	39	31.97	60	25	7	15	18	2.09 (0.94)
	Raters	4	31.50	100	0	100	0	0	2.26 (0.82)
	Coders	4	44.25	75	100	50	100	100	3.50 (0.88)

1. *Writers* were 166 healthcare professionals (practitioners, researchers and research students) with an interest in investigating, reviewing and designing or delivering BCIs but with little or no previous knowledge of using BCTTv1. We considered this group to be appropriate given their expressed interest and/or experience in BCIs; they reflect the wider population of healthcare professionals who would report the content of interventions as part of their current or future employment. All writers had signed up to complete a BCTTv1 training workshop and participated in this study during the workshop. Six workshops were delivered by two or three members of the BCTTv1 project team. They were held at Newcastle University (*N* = 26), the University of Oxford (*N* = 16), Queens University Belfast (*N* = 45), the University of Aberdeen (*N* = 24), University College London (*N* = 15) and the University of Manchester (*N* = 16). Workshops were advertised via scientific and professional organisations and via the BCTTv1 project website (http://www.ucl.ac.uk/health-psychology/bcttaxonomy).
2. *Raters* (*unfamiliar with BCTTv1*) were 12 healthcare professionals and trainees unfamiliar with BCTTv1. As the interventions used in our studies targeted smoking cessation interventions, we recruited raters from the National Centre for Smoking Cessation and Training (NCSCT) database (www.ncsct.co.uk): an online training resource for smoking cessation advisors to support delivery of smoking cessation interventions. Raters were required to be unfamiliar with BCTTv1 as they were asked to rate the quality (in terms of clarity and replicability) of the written descriptions. Thus, we only invited those who had registered with the NCSCT but who had not yet commenced their training. Raters familiar with BCTTv1 would be inclined to judge the quality based on the presence/absence of BCTs in the description using identical terms as in BCTTv1 which was not the objective of the task.
3. *Coders* (*familiar with BCTTv1*) were 12 behaviour change researchers and practitioners, previously trained to code BCIs using BCTTv1 via distance group tutorial training [9, 16]. All coders had demonstrated an acceptable level of competence in using BCTTv1 to reliably and validly identify the content of BCIs i.e. they achieved a good level of agreement with experts in a formal BCT coding assessment [16].

### Procedure

**Fig. 1:**
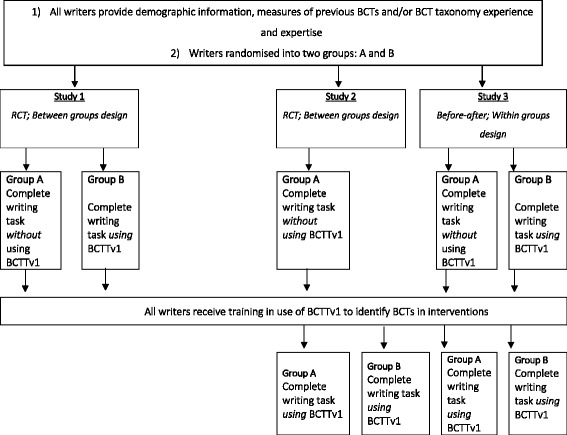
Design and procedure for studies 1, 2 and 3

Figure 1 presents details of study procedures. At the beginning of each workshop, writers completed a questionnaire with demographic information (i.e. age, gender, nationality, professional background, highest qualification) and measures of previous BCTs and/or BCT taxonomy experience and expertise. Writers were randomised into two groups by selecting a letter (either A or B) from a bag containing pieces of paper that were marked with equal numbers of As and Bs. The total number of letters in the bag was equal to the number of writers. Writers were thus randomised into groups (A and B) to determine which writers would complete the writing task using BCTTv1 and which writers would complete the writing task without BCTTv1. Writers provided with BCTTv1 for the task completed measures of its usefulness and acceptability. All writers then completed training in the use of BCTTv1 to identify BCTs in interventions. The workshops included three short PowerPoint presentations and the writers participated in a series of interactive coding tasks as a group, individually and in pairs. The workshop was structured around a series of learning objectives and the BCTs delivered as part of the training sessions aimed to improve behavioural performance (e.g. behavioural practice/rehearsal, feedback on behaviour, graded tasks). The workshop and training materials are described in more detail elsewhere (Wood et al. 2014). As an initial evaluation of this novel task, at the end of the workshop the writers were asked to rate the usefulness of BCTTv1 for reporting BCIs.

#### Writing task

Writers watched a video of a smoking cessation intervention being delivered (described above under ‘Materials’ section). The video was shown once and writers were encouraged to write notes about the ‘active content’ (i.e. content delivered that was likely to change behaviour) during the video. Writers were given 15 min to write a description in such a way that (a) the active content being delivered in the intervention could be understood and (b) the intervention could be replicated by someone else. Dependent on group allocation, some writers were provided with a copy of BCTTv1 for the task whilst others were not. Writers who received BCTTv1 were given an additional 5 min at the start of the task to familiarise themselves with the taxonomy (i.e. briefly read labels, definitions and examples). Workshop facilitators monitored participants during these 5 min to ensure that they did not start the writing task during these 5 min.

#### Rating written descriptions

Raters were sent materials via email and could complete and submit their ratings remotely. They completed the same demographic questionnaire as the writers (see ‘Measures’ section). They were randomly allocated a set of written intervention descriptions (the number of descriptions in the set differed according to study) stratified so that sets comprised an approximately equal number of descriptions written about video 1 and video 2. Raters were instructed to read each description carefully and rate it according to (a) ease of understanding and (b) adequacy of information required to undertake a replication (see ‘Measures’ section).

#### BCT coding of written descriptions

Using the same randomisation methods as described for raters, coders were randomly allocated a set of written intervention descriptions. They could complete and submit the task remotely. The coders were asked to identify BCTs using BCTTv1 (see ‘Measures’ section). The coding was estimated to take up to one day. Invitations were sent with an offer of an honorarium of £280 on completion of the coding.

### Measures

Measures included the following: previous experience and BCT taxonomy expertise (writers, raters and coders) - previous experience in (a) designing or reporting BCIs that specifically identified BCTs, (b) writing manuals or protocols of interventions and (c) undertaking a narrative or systematic review of behaviour change literature; expertise (i.e. knowledge, skills and familiarity) in (a) designing or reporting BCIs that specifically identified BCTs; (b) involvement in writing manuals or protocols of interventions; (c) undertaking a narrative or systematic review of behaviour change literature. All used response options from 1 (‘no experience’) to 5 (‘a great deal of experience’).

#### Quality of intervention descriptions (raters)

Each description was assessed by two raters. For each description, raters used four scales from −3 (strongly disagree) to +3 (strongly agree) to indicate agreement with

1. ‘I can clearly visualise how the intervention was delivered’
2. ‘I can clearly visualise what was delivered in the intervention’
3. ‘Someone would be able to replicate how the intervention was delivered’
4. ‘Someone would be able to replicate what was delivered in the intervention’

#### Identification of BCTs described (coders)

Each description was coded by two coders. Coders used BCTTv1 to identify which BCTs were absent and which were present in each of the written descriptions. Agreement of BCT identification was calculated between pairs of coders to assess inter-rater reliability: the extent to which the two trained coders can apply BCTTv1 consistently to identify BCTs. We also calculated agreement between the coders and consensus about the BCTs present and absent in the smoking cessation interventions by the team who developed BCTTv1. This aimed to assess validity: the extent to which the two trained coders can apply BCTTv1 accurately and identify the same BCTs as the developers of the taxonomy. It is important to assess validity in addition to reliability [9]. Both measures of agreement were calculated using the prevalence and bias-adjusted Kappa statistic (PABAK; [17]).

#### Usefulness and acceptability of using BCTTv1 (writers)

Using a scale from 1 ‘strongly disagree’ to 7 ‘strongly agree’, writers rated whether the time allocated for the writing task was sufficient. They rated the difficulty of the writing task from 1 ‘very easy’ to 7 ‘very difficult’. Those provided with BCTTv1 for the writing task rated

1. ‘Using BCTTv1 to describe the content of BCIs is…’ using 7-point bipolar adjective scales (i.e. pairs of opposites):
  Writers rated (using a scale from 1 ‘strongly disagree’ to 7 ‘strongly agree’):
  ○ Difficult–easy
  ○ Useful–worthless
  ○ Good–bad
  ○ Undesirable–desirable
2. ‘If I use BCTTv1 to describe the content of behaviour change intervention…’
  ○ ‘…my description will be clear’
  ○ ‘…someone will be able to replicate the intervention after reading my description’

### Analysis

Mean ratings and mean PABAK scores were entered as dependent variables into separate analysis of variance. A 2 (video 1 vs. video 2) × 2 (written with BCTTv1 following training vs. written without BCTTv1 prior to training) factorial design was used for studies 1 and 2; a repeated measures design was used for study 3. Mean ratings were calculated to describe writers’ evaluation of using BCTTv1.

## Results

A total of 166 BCI descriptions were generated across the three studies.

- Study 1: 42 descriptions written prior to training; 24 written without BCTTv1 and 18 written with BCTTv1
- Study 2: 85 descriptions; 29 written without BCTTv1 prior to training and 56 written with BCTTv1 after training
- Study 3: 78 descriptions; 39 written without BCTTv1 prior to training and 39 written by the same writers, with BCTTv1 and after training

Research question (RQ) 1: Are descriptions of observed BCIs generated by untrained writers using BCTTv1 (i) clearer and (ii) more replicable than descriptions written by untrained writers not using BCTTv1?

Means and standard deviations for all the dependent variables are summarised in Table 2.

**Table 2:** Means for all measured variables across Studies 1, 2 and 3 (standard deviations given in brackets)

			Study 1		Study 2		Study 3	
			Effect of using BCTTv1 (between-group)		Effect of training and using BCTTv1 (between-group)		Effect of training and using BCTTv1 (within-group)	
Research question		Video	Untrained + no taxonomy (*n* = 24)	Untrained + taxonomy (*n* = 18)	Untrained + no taxonomy (*n* = 29)	Trained + taxonomy (*n* = 56)	Untrained + no taxonomy (*n* = 39)	Trained + taxonomy (*n* = 39)
1	‘I can clearly visualise what was delivered in the intervention’		0.83 (1.44)	1.47 (1.18)	1.76 (0.62)	0.88 (1.07)	−0.13 (1.20)	0.64 (1.48)
		1	1.75 (0.60)	0.94 (1.40)	1.71 (0.82)	0.55 (1.19)	0.26 (1.09)	0.83 (1.52)
		2	0.38 (1.53)	1.90 (0.81)	1.45 (0.64)	0.51 (1.23)	−0.53 (1.21)	0.45 (1.44)
	‘I can clearly visualise how the intervention was delivered’		1.02 (1.37)	1.53 (1.16)	1.14 (1.14)	0.14 (1.09)	−0.17 (1.19)	0.59 (1.61)
		1	1.94 (0.62)	0.94 (1.18)	1.00 (1.03)	0.00 (1.26)	0.29 (1.14)	0.68 (1.73)
		2	0.56 (1.42)	2.00 (0.94)	0.70 (1.64)	−0.42 (1.05)	−0.63 (1.04	0.50 (1.53)
	‘Someone would be able to replicate what was delivered in the intervention’		0.69 (1.56)	1.39 (1.30)	1.64 (0.76)	1.02 (0.86)	−0.18 (1.22)	0.40 (1.50)
		1	1.56 (0.42)	0.87 (1.41)	1.53 (0.92)	1.05 (1.09)	0.16 (1.07)	0.38 (1.39)
		2	0.25 (1.74)	1.80 (1.11)	1.55 (0.69)	0.81 (0.81)	−0.53 (1.29)	0.43 (1.63)
	‘Someone would be able to replicate how the intervention was delivered’		0.69 (1.56)	1.42 (1.10)	1.31 (0.88)	0.47 (1.04)	−0.25 (1.17)	0.45 (1.47)
		1	1.50 (0.71)	1.00 (1.10)	1.18 (0.92)	0.43 (1.20)	0.11 (1.14)	0.45 (1.41)
		2	0.28 (1.72)	1.75 (1.03)	1.05 (0.93)	0.28 (1.01)	−0.61 (1.11)	0.45 (1.56)
2	Reliability of BCT identification (PABAK between coders)		0.86 (0.05)	0.88 (0.05)	0.84 (0.07)	0.87 (0.06)	0.85 (0.06)	0.86 (0.06)
		1	0.87 (0.06)	0.88 (0.06)	0.84 (0.05)	0.85 (0.04)	0.86 (0.06)	0.85 (0.06)
		2	0.86 (0.05)	0.88 (0.05)	0.84 (0.05)	0.83 (0.09)	0.85 (0.06)	0.85 (0.06)
3	Validity of BCT identification (PABAK between coders and developer consensus)		0.69 (0.05)	0.69 (0.06)	0.67 (0.05)	0.70 (0.05)	0.67 (0.06)	0.66 (0.06)
		1	0.65 (0.03)	0.65 (0.07)	0.66 (0.04)	0.69 (0.05)	0.63 (0.04)	0.61 (0.04)
		2	0.72 (0.04)	0.70 (0.05)	0.66 (0.04)	0.72 (0.05)	0.72 (0.03)	0.71 (0.04)
4	Sufficiency of time allocated for task	-	5.43 (1.74)	5.56 (1.32)	6.06 (1.43)	4.90 (1.41)	5.36 (1.46)	4.20 (1.90)
	Difficulty of writing task	-	4.71 (0.73)	4.88 (1.03)	4.72 (1.27)	4.30 (1.22)	5.00 (1.03)	4.80 (0.68)
	Paired adjectives							
	Difficult vs. easy	-	4.25 (0.89)	3.56 (1.32)	4.40 (0.89)	3.75 (1.25)	3.86 (1.41)	3.38 (1.31)
	Worthless vs. useful	-	6.50 (0.53)	6.25 (0.93)	6.00 (1.00)	4.70 (2.08)	6.43 (0.65)	6.00 (1.43)
	Bad vs. good	-	6.75 (0.46)	6.25 (0.86)	6.00 (0.82)	4.60 (1.98)	6.29 (0.61)	6.41 (1.04)
	Undesirable vs. desirable	-	6.50 (0.76)	5.31 (2.00)	6.40 (0.89)	5.85 (1.04)	5.86 (1.29)	6.38 (0.71)
	Description will be clear	-	5.70 (1.49)	5.20 (1.57)	5.67 (0.52)	5.68 (1.00)	6.21 (0.80)	5.54 (1.35)
	Description will be replicable	-	5.70 (1.83)	5.07 (1.49)	5.50 (1.05)	5.37 (1.26)	6.07 (0.92)	5.49 (1.30)

For research questions 1 and 2, all items had response options from −3 ‘strongly disagree’ to +3 ‘strongly agree’; for research question 3, all items had response options from 1 ‘strongly disagree’ to 7 ‘strongly agree’

In study 1, using BCTTv1 compared to not using BCTTv1 (both without training) failed to improve description quality in terms of: clarity of active ingredients, *F*(1,38) = 0.82, *p* = 0.37, clarity of mode of delivery, *F*(1,38) = 0.35, *p* = 0.56, replicability of active ingredients, *F*(1,38) = 0.96, *p* = 0.33, or replicability of mode of delivery, *F*(1,38) = 1.31, *p* = 0.26.

RQ2: Are descriptions of observed BCIs generated by trained writers using BCTTv1, (i) clearer and, (ii) more replicable than descriptions written by untrained writers not using BCTTv1?

Study 2 shows that compared to no training, training in the use of BCTTv1 to specify BCTs decreased the quality of descriptions: clarity of active ingredients, *F*(1,81) = 16.09, *p* < 0.001, clarity of mode of delivery, *F*(1,81) = 14.08, *p* < 0.001, replicability of active ingredients, *F*(1,81) = 8.05, *p* < 0.01, replicability of mode of delivery, *F*(1,81) = 9.62, *p* < 0.005.

In study 3, descriptions written using BCTTv1 and following training were rated as being of higher quality than descriptions written by the same writers without using BCTTv1 and before training. We found significant effects on all measures apart from replicability of active ingredients, *F*(1,74) = 3.57, *p* = 0.06. Significant findings were for clarity of active ingredients, *F*(1,74) = 6.51, *p* < 0.05, clarity of mode of delivery, *F*(1,74) = 5.71, *p* < 0.05, and replicability of mode of delivery, *F*(1,74) = 5.46, *p* < 0.05.

RQ3: Does the reliability of BCT identification differ for descriptions of observed BCIs written by (i) untrained writers not using BCTTv1, (ii) untrained writers using BCTTv1 and, (iii) trained writers using BCTTv1?

In study 1, using BCTTv1 without training did not improve the identification of BCTs. Agreement between coders was similar for descriptions written prior to training, with and without using BCTTv1, *F*(1,38) = 0.01, *p* = 0.93.

Study 2 indicates that training in the use of BCTTv1 resulted in descriptions from which BCTs could be coded more reliably. Agreement between coders was greater for descriptions written by writers with BCTTv1 following training than for descriptions written by those without BCTTv1 prior to training, *F*(1,81) = 5.02, *p* < 0.05.

Study 3 did not replicate this finding from study 2. Agreement between coders did not differ significantly for descriptions written before training, without using BCTTv1 and for descriptions written by the same writers, after training and using BCTTv1, *F*(1,74) = 0.46, *p* = 0.50.

RQ4: How does coder coding compare to that of developer consensus coding?

There were no effects of using BCTTv1 or BCTTv1 training on coder agreement with BCTs identified by those who developed BCTTv1 (all three studies *p*s > 0.05).

RQ5: Do users report BCTTv1 to be a useful and acceptable tool for reporting the content of BCIs?

Table 2 shows means and standard deviations from all measures. The writers reported that using BCTTv1 to report BCTs was a challenging task across all conditions and studies: *M =* 3.86, SD = 0.39. Across all studies and conditions, writers reported that using BCTTv1 to report BCIs was a ‘useful’ (*M =* 5.98, SD = 0.66), a ‘good’ (*M =* 6.05, SD = 0.75) and a ‘desirable’ method (*M =* 6.05, SD = 0.46) and that written intervention descriptions would be clear (*M =* 5.67, SD = 0.33) and replicable (*M =* 5.53, SD = 0.33) if BCTTv1 was used in practice.

## Discussion

In studies 1, 2 and 3, we investigated the effect of using BCTTv1 alone, and the effect of BCTTv1 plus training, on describing observed interventions, using randomised between-group and before-after, within-group designs. We found no benefit of providing BCTTv1 in terms of clear and replicable reporting observed intervention descriptions. The descriptions generated by writers with BCTTv1 following training performed better than those of the control condition only for the reliability of identifying BCTs (study 2) and for the clarity and replicability of the descriptions (study 3). The results of study 3 might simply be due to practice effects as writers had time to improve their writing skill during the workshop. Furthermore, baseline scores in study 3 were much lower than for the comparable group in study 2, so there was more room for improvement.

Untrained writers produced descriptions which were rated to be of higher clarity and replicability than trained writers in study 2, where participants were randomised and no practice effects were possible. These results are unexpected and difficult to explain. Although BCTTv1 appears to have made descriptions easier to recognise in one study, it appears to have done the opposite in another. There are six possible explanations for the results of study 2:

1. Trained writers completed the task at the end of a workshop and may have been more tired than untrained writers completing the task at the beginning of the day. If so, then one might expect their descriptions to be shorter and less detailed. However, this result was not found in study 3, where all writers completed the writing task at the beginning and at the end of the workshop.
2. Trained writers may have written longer or more complex descriptions which made it more difficult for coders to identify BCTs.
3. Trained coders may have concentrated on reporting BCTs and omitted other descriptive elements such as detail about the delivery procedures which might render the description clearer to understand and therefore judged to be more replicable.
4. The time constraint may have seemed more restrictive for the trained than the untrained writers with the result that their descriptions were less clear. The demands of the task may have seemed especially high to trained writers, expected to implement new knowledge and skill from their training to use the taxonomy with 93 BCTs in addition to reporting the content of a 9-min video, which was shown only once.
5. Immediately following training, writers may be unable to clearly report all of their ideas about the intervention and may need more experience with BCTTv1 (e.g. familiarity with BCT labels and definitions, ability to locate and select BCTs from the taxonomy with speed and confidence and distinguish between similar BCTs) to write good descriptions.
6. The training focused on identifying BCTs from written descriptions and videos. Only 30 min of the workshop was devoted to how to write sentences about active ingredients using BCTTv1 and was accompanied by a single task. This is a challenging task that requires interpretation of content observed in the video. Additionally, no emphasis was placed on the importance of writing down other intervention components, e.g. delivery procedures, alongside BCTs. The training in this sense could have been insufficient to improve skill in reporting all intervention content.

These explanations can be tested by examining whether controlling for the following variables alters the findings: the number of words (explanations 1 and 2), the intelligibility of the text (explanation 2), the number of BCTs identified (explanation 3) and ratings of time pressure and difficulty (explanation 4). Testing explanation 5 would require a longitudinal study, and explanation 6, the development and evaluation of an optimised training package for using BCTTv1 to report BCTs.

There were limitations in the design, the measures and the writing task. In terms of design, the studies confounded training condition with time of day, which was inevitable given the resources of the study. We could have asked participants to write the descriptions at a later date at the same time of day, although this introduces the possibility of further confounders such as additional reading by either group.

In terms of measures, we used indicators that reflected the likelihood that readers of the descriptions would be able to visualise the intervention in such a way that they felt confident about being able to replicate it. Whilst there are guidelines which aid the assessment of whether or not intervention components are reported, the standardised measures for assessing the *quality* of reporting of active ingredients and mode of delivery in intervention descriptions are still developing (e.g. Hoffman et al. 2014; Chan, Gotzsche et al. 2013). Hence, we developed measures of clarity and replicability specifically for studies 1, 2 and 3 and ensured that active content was rated separately from mode of delivery of the intervention. However, further work is required to establish their psychometric properties. Whilst the use of indices of reliability of BCT identification is an accepted method, assessing validity of BCT identification is less well established. We used consensus following independent judgments by the experienced BCTTv1 project team (the ‘developers’) as our best approximation to a criterion of validity against which judgments could be evaluated.

The writing task was unusual and differed from normal practice in intervention development and reporting. Participants were given a restricted amount of time, which we considered to be appropriate for the complexity of the task. In normal practice, intervention reporters would describe their own intervention not by observing it on a video but by referring to intervention materials and study procedures. The task was constrained by the time available within the full-day workshop. It was not feasible to increase the duration of the workshop, which incorporated other didactic and interactive components. It was challenging to decide on the appropriate time for the task as this is the first evaluation of using BCTTv1 to report behaviour change interventions. Nevertheless, participants did not express dissatisfaction with the task in their feedback. However in retrospect, a better test of BCTTv1 for this purpose would be to investigate whether the use of BCTTv1 *enhances* existing intervention descriptions rather than using it to create descriptions simply from observation. For example, authors of published intervention protocols might be invited to re-write the intervention description with or without BCTTv1 and research participants asked to choose the ‘better’ description from each pair of the new descriptions.

The finding of unexpected negative effects of training is consistent with some of the counter-intuitive findings reported in the psychology literature. For example, therapists who participated in a training programme for professional psychotherapy (manual-based and involving didactic seminars and small-group supervision) showed unexpected deterioration in skills after training [18]. The authors proposed that this was evidence of a ‘post-training phase in which [participants’] performance actually declined in certain ways as they struggled to naturally integrate new techniques into their existing styles and approaches’ (p. 439). There is evidence of a paradoxical effect of incentives on skilled performance, described as a ‘choking effect’ [19]. That is, striving to improve performance following training increases pressure on the participant and can lead to poorer performance. Furthermore, participants under high mental load and in a stressful situation displayed greater levels of physiological stress reaction when asked to relax than participants who were not asked to relax [20]. It is possible that the participants in our study similarly responded to the intervention writing task in ways that parallel a choking effect or the post-training effect described in these examples. Hence, it may be that measurable benefits of training occur after a period of integration and practice. Further follow-up research could explore this possibility.

## Conclusions

In conclusion, we did not find benefits of providing a copy of BCTTv1 for reporting observed interventions in a time-pressured situation. This may be due to task demands, the nature of the task evaluated, omission of other intervention components, lack of time and experience or an insufficient training package. Further work is needed to improve its utility in this area. This includes further examination of the data to identify potential reasons for the unexpected findings, testing the utility of BCTTv1 in enhancing existing intervention descriptions, and potential improvements of the training package, such as more detailed guidance for how to use BCTTv1 to report the active content of interventions alongside mode of delivery, and a wider range of interactive tasks to train users in reporting good quality descriptions. Further work is also needed to train users in how to use BCTTv1 to report descriptions of newly developed interventions. Until such work has been done, we can make no recommendations about using BCTTv1 to report interventions.

## Abbreviations

ANOVA, Analysis of Variance; CONSORT, Consolidated Standards of Reporting Trials; BCI, Behaviour Change Intervention; BCT, Behaviour Change Technique; BCTTv1, Behaviour Change Technique Taxonomy Version 1; NCSCT, National Centre for Smoking Cessation and Training; PABAK, Prevalence- and Bias-Adjusted Kappa; RQ, Research Question; SD, Standard Deviation; SPIRIT, Standard Protocol Items: Recommendations for Interventional Trials; TIDieR, Template for Intervention Description and Replication checklist

## Additional file

Additional file 1: Transcripts of Video 1 and Video 2. (DOCX 20 kb)
