# Supplementary material for: Reporting behaviour change interventions: do the behaviour change technique taxonomy v1, and training in its use, improve the quality of intervention descriptions?
Source: Implement Sci. 2016 Jun 7;11:84. doi: 10.1186/s13012-016-0448-9 (PMC4897953; doi:10.1186/s13012-016-0448-9)
Supplement: Additional file 1: — Transcripts of Video 1 and Video 2. (DOCX 20 kb) [file 13012_2016_448_MOESM1_ESM.docx]

**ADDITIONAL FILE 1**

1. **Transcript of Video 1**

Key: (1) Practitioner, (2) Patient

Target Behaviour: Smoking cessation

| 1 | If I can just explain what coming to an NHS stop smoking service involves. I’ll see you once before your quit date which is now obviously, we’re then going to decide a date suitable for you which is going to be your quit date [Goal setting (behaviour); Action planning]. I’ll then see you for four weeks after that weekly, ok? [Social support (unspecified)]. Four weeks after your quit date then you’ll stop seeing me, but you’ll still have the medication [Pharmacological support] and the motivation you’ve built up to keep you going ok? Eight to twelve weeks after the quit date we’re going to reduce the amount of medication rather than stopping completely and that’s kind of a nice way of gradually reducing that support that you will be getting [Graded tasks]. You will find you need most of that support within the first four weeks after the quit date which is why you’ll be coming to see me. Now… the benefit of seeing someone such as myself who is trained as a stop smoking specialist means it roughly doubles your chances of stopping smoking permanently [Credible source] and then using the medication with that also roughly doubles your chances so you’re definitely in the right place. |
| --- | --- |
| 1 | Now when people start smoking, their brain actually undergoes physical changes. Now minds and bodies get really used to regular doses of nicotine. When they stop smoking they get withdrawal symptoms [Information about social and environmental consequences] which I’m sure you know about… |
| 2 | Yeah |
| 1 | …they get strong urges and cravings to smoke. Now there are other symptoms which you may have experienced, where you’ve maybe gone a little while without being able to have a cigarette, ok. Now these urges and cravings are the thing which can really get people down [Information about emotional consequences]. You know, ruin their attempt at quitting ok. Now… thankfully there are medications which can help with this. Ok? [Problem solving]. What I need to do is to find out how heavily dependent you are on cigarettes just so I can establish what kind of medication you’re going to need [Body changes]. So can I just ask you roughly how many cigarettes do you smoke a day? |
| 2 | Um… around twenty a day? Depends on the day I guess so it can be as… twenty-five to thirty? |
| 1 | Mainly at the weekends is that? |
| 2 | Yeah mostly, yeah. |
| 1 | Ok. And when you wake up in the morning how soon do you find you have your first one? |
| 2 | Pretty much straight away |
| 1 | Within half an hour? An hour? |
| 2 | Yeah, I’d say within about ten minutes. |
| 1 | Ok within ten minutes ok right. Well, from what you’ve said from having between twenty and thirty a day and having your first cigarette so soon after waking up, I would say you’re quite heavily dependent on cigarettes so it is great that you’ve come here to see us. You are going to need quite a high dose medication but we’ll come on to that in a minute and I’ll tell you all about that. |
| 2 | Ok. |
| 1 | Now Jack, every time you see me we’ll do a simple carbon monoxide test and you may already know this. Carbon monoxide is a poisonous gas that’s found in the smoke you inhale from your cigarettes ok? And it replaces some of the oxygen in your blood and that can lead to coronary heart disease. Ok. At this point I would expect your carbon monoxide levels to be fairly high because you are still smoking, and the same will apply on your quit date as well. Probably be expecting something above ten parts per million. Ok? Now, below ten parts per million is something we would call a non-smoker. And that’s normally in the range of two to six parts per million. Now the good news is when you do come back after your quit date, as long as you haven’t smoked at all I would expect it to be the same as a non-smoker. [Incentive (outcome)] Ok? |
| 2 | Yeah |
| 1 | Basically, we do the test for two reasons. The first is if you haven’t smoked, you’ll be able to see straight away that your carbon monoxide levels have gone down to that of a non-smoker and you can start to see the benefits of that immediately. But secondly, if you have smoked we are going to be able to know about it [Monitoring outcome of behaviour by others without feedback; Biofeedback] so you can’t actually get away with smoking |
| 2 | (laughs) ok |
| 1 | now Jack, what I’m going to ask you to do in a minute is to hold your breath, take a big deep breath and just hold it for fifteen seconds, ok? Now, sometimes when I tell people that they seem a bit alarmed. It does seem like a long time but you’ll be absolutely fine ok? Now, what I’m going to do is to count you down and in the last ten seconds I’m going to hand this machine to you. It’s a carbon monoxide monitor. I’m going to hand that to you and then after it counts down it will beep for the last three seconds, three, two, one and I’ll say ‘exhale’, ok? You’re going to make a nice tight seal around the tube there and just let all of the air come out of your lungs. You don’t have to blow too hard or anything. And then we’ll just get a nice reading from you ok? |
| 2 | Ok |
| 1 | brilliant ok. So, take a nice big deep breath for me ok? Excellent, and hold it…Ok, ten seconds left. I’ll give you that (hands monitor to client)…and three, two, one…and exhale. Brilliant, ok excellent (client hands monitor to advisor). Well, as I said to you before, the typical range for a non-smoker is somewhere between two and six parts per million. So, as you can see here with yours its thirty-five parts per million [Behavioural experiments]. It’s about seven to eight times that of a non-smoker. There’s no need to worry about that. It’s what we would expect as you are still smoking. The excellent news for you is that the next time we do this after your quit date, what you’ll find is that it will go down to somewhere between two and six. You’ll have exactly the same reading as a non-smoker ok? |
| 2 | brilliant, right |
| 1 | So Jack, having explained the reasons for not even a puff after your quit date, what I really want now is to hear you saying it instead of me saying it that you’re prepared to put in the effort to not even have a single puff after that quit date. So can you do that? [Commitment]. |
| 2 | Yeah, yeah absolutely |
| 1 | Can you say that…now…to me? |
| 2 | You want me to say to you now…that I won’t… |
| 1 | Yes please, yeah |
| 2 | Right ok. I will not have a cigarette at all after my quit date. |
| 1 | Brilliant. See. Well done. |
| 1 | In the first week you are going to experience frequent strong urges to smoke again. So it’s really important to make sure you get all temptation out of the way. What we suggest is that you go through your coat pockets, your drawers and anywhere else that you might have cigarettes and obviously get rid of them and obviously things like ashtrays and lighters and all that kind of stuff you just not going to need it any more. So if you clear all that away from you. Obviously you said that your girlfriend does smoke, she’s not giving up but she’s supporting you with your quit attempt. It’s really important that she keeps all of her smoking paraphernalia out of the way as well and that she doesn’t smoke around you. [Avoidance/reducing exposure to cues for the behaviour; Restructuring the social environment; Restructure the physical environment; Social support (practical)]. Ok? |
| 2 | Ok |
| 1 | So Jack, it’s the end of sessions now. You’ve done ever so well [Social support (unspecified)] and you know that yourself. You’ve stopped smoking completely for four weeks and that’s brilliant [Social reward; Feedback on behaviour]. How are you feeling now about the future? |
| 2 | Yeah. Quite positive, I mean it’s been quite good coming down here and I obviously know I cannot smoke and I’ve been tested, I quite like being checked up on. Something to aim for and it’s quite nice to have that. And to be completely honest I would quite like to have another couple of sessions quite frankly |
| 1 | Well it kind of works out quite naturally that you’re going to have to stop seeing me at this point as you’ve had that support in the first four weeks where the withdrawal symptoms are really strong. And now it’s more over to you and the motivation you’ve built up and the medication ok? I’m going to be writing a letter to your GP, just to ensure that you are still going to be getting that medication as it’s really important that you keep that up [Social support (practical)]. You’ve also got the option where if you do feel like you’re panicking or you’re really at risk of going back then give us a call and you can get an emergency appointment if it’s really critical ok? I’m going to give you a number for the NHS Stop Smoking Helpline [Problem solving]. If you do feel like you’re at risk of lapsing again then you can call them and from seven in the morning till eleven at night they’ll be there for someone for you to talk to ok [Social support (unspecified)]. But I’m confident now that you have every opportunity of becoming a permanent non-smoker. If anything does go wrong at any point in the future, don’t be frightened of coming back. You won’t be the first person that we’ve seen that happen to. What we’re really interested in is you becoming a permanent non-smoker. You’ve done ever so well so far so congratulations and good luck for the future |
| 2 | thanks very much |

1. **Transcript of Video 2**

Key: (1) Practitioner, (2) Patient

Target Behaviour: Smoking cessation

| 1 | Hi…Lizzy I’m Angela, it’s great to see you here. Take a seat. Right, it’s great to see you here. Now…I know that you want to stop smoking. Um, but can I just ask you first of all why you do want to stop smoking? What your reasons are? |
| --- | --- |
| 2 | I know it’s not good for me and um… and I’ve been smoking a really long time and I’ve tried to give up before and it didn’t really work out. And I think if I don’t really do it now then it’s never going to happen. And I’ve got kids. |
| 1 | Ok. Well it…as I said it’s good that you’re here. And many smokers do want to stop smoking but not that many make a quit attempt. And even fewer come to get help from a stop smoking service so it’s good that you are here. And stopping smoking is good for your children as well um, as you probably know [Information about social and environmental consequences; Information about health consequences]. They won’t see you smoking so they’re less likely to become smokers themselves [Identification of self as role model]. Can I just check that you are willing to give up smoking and to give up completely [Commitment] because that’s what this programme is about? |
| 2 | Yeah (coughs) I want it out of my life now |
| 1 | Ok that’s great. Right, now… you’re still going to have to put a considerable effort into stopping smoking even with the help of the stop smoking service and the medication. |
| 2 | Yeah alright |
| 1 | Ok. Great. |
| 1 | Well Lizzy, is there anyone who lives with you who smokes? Any smokers at work or friends of yours? |
| 2 | Yeah… quite a few actually. Well, my husband… he smokes, quite heavily even more than I do. Erm… and at work there are a few people who smoke although a few people have given up. And my friends… less smoke now than used to but there are still quite a few. |
| 1 | Ok and your husband, I don’t suppose he wants to give up by any chance does he? |
| 2 | (shakes head) No. |
| 1 | Well as you probably know, you are going to want a cigarette after your quit date and the urge to smoke is probably going to be quite strong at times [Information about emotional consequences]. So you really don’t want to see people smoking around you or be exposed to cigarettes as it is going to make it difficult for you. So it’s best if you can limit your exposure to cigarettes [Restructuring the social environment] especially in the first few weeks when it’s at its toughest. So what you want from people at work and your husband at home is for them not to smoke in front of your or not to see their cigarettes lying around [Restructuring the physical environment; Avoidance of exposure to cues] because it really will make it difficult for you. |
| 1 | So Lizzy, are there people out there who you’ll be able to turn to for help and support? [Social support (unspecified)] |
| 2 | Yes. Err… there’s a few people at work who have given up recently so I know they’ll be really supportive of me stopping. Obviously, my children are desperate for me to stop and err… my husband even though he doesn’t want to give up himself, he knows how much I want to and he’s… supportive of that |
| 1 | Well that’s great. I mean your children especially will be a reminder of why you want to give up in the first place. And it’s great that you’ve got friends at work that have been through this process and will really understand what you’re going through and will be supportive. And it’s great too that your husband is willing to be supportive and will be able to help you [Social support (unspecified)]. Now what you need from him first of all is for him not to smoke in front of you and for him not to leave his cigarettes lying around as that really will be a dangerous thing for you. |
| 1 | So Lizzy, it’s great to see you again. Now, first of all do you want to tell me how you got on since your quit date last week? |
| 2 | Er… yeah really good actually because you know how I’ve been smoking twenty, maybe up to thirty cigarettes a day, I’ve managed to cut down to like, five cigarettes a day. And um…it’s just so much less that I was smoking before so it works out at like thirty a week rather than thirty a day so I’m really, really happy about that. |
| 1 | Uh, so obviously that’s a big difference from your smoking levels before but do you remember when we discussed the importance of stopping smoking completely after your quit date [Feedback on behaviour; Goal setting (behaviour)]. Now, it seems to me you had a change of plan there and go for less smoking rather than no smoking at all [Discrepancy between current behaviour and goal]. So can you just talk me through why you changed your mind on that point? |
| 2 | Um… yeah I didn’t really change my mind really but it was just too hard to quit smoking altogether so I thought it was a lot better for me to cut down rather and just wean myself off. |
| 1 | Ok I can understand that and this has happened to other smokers at this early stage, and that’s something I think we can talk about and try and discover what it was on that first day that meant that you weren’t able to go through with it and completely stop smoking as you’d planned to. Now it might be a matter of looking at your medication, you might need some help with that. But we can look a bit more closely at what actually happened on that first day that meant you weren’t able to get your head around stopping smoking completely |
| 1 | So Lizzy, this is the third week after your quit date. It’s great to see you back, how have you been this week? |
| 2 | To be honest, not great. It’s been alright in terms of the not smoking, I haven’t smoked at all. But I’ve found this week really, really hard which I wasn’t expecting. I was expecting thing to get better but it actually felt like it got worse. And I just feel a bit worn down by trying to sort of constantly motivated to keep myself off the fags |
| 1 | Well I’m going to give you some congratulations first of all because even though you’ve been feeling down you haven’t smoked, that’s three weeks without a cigarette so well done for not smoking [Social reward]. And this will give you a really good chance from moving on from that. Now despite the fact that it’s been difficult for you, you’ve managed to overcome that. It’s not uncommon for smokers to be fuelled by motivation and the help and support from friends and family. And that wanes after a bit so the third and fourth week can seem a bit of a slog [Information about emotional consequences]. Now the other thing which can happen to smokers, I know you’ve been using the patches and lozenges, is that they use less medication than they should. Is that what you’ve been doing? |
| 2 | Uh… actually maybe yes I have been using less lozenges yeah. |
| 1 | Ok, well if you’re getting quite severe withdrawal symptoms like a really strong urge to smoke and a low mood [Information about emotional consequences] then I would suggest that you’re probably not using enough nicotine replacement therapy. But you can get over this. You’ve been through a really difficult week already. Ok so you can do it. So I would suggest that you make a plan to use a little bit more nicotine replacement therapy [Pharmacological support; Action planning] this week and that will really help. |
